# Development and Characterization of PEGDA Microneedles for Localized Drug Delivery of Gemcitabine to Treat Inflammatory Breast Cancer

**DOI:** 10.3390/ma15217693

**Published:** 2022-11-01

**Authors:** Ahmed Alafnan, Aravindram Attiguppe Seetharam, Talib Hussain, Maram Suresh Gupta, Syed Mohd Danish Rizvi, Afrasim Moin, Abdulwahab Alamri, Aziz Unnisa, Amir Mahgoub Awadelkareem, AbdElmoneim O. Elkhalifa, Pradyumna Jayahanumaiah, Mohammad Khalid, Natchimuthu Balashanmugam

**Affiliations:** 1 Department of Pharmacology and Toxicology, College of Pharmacy, University of Ha’il, Ha’il 81442, Saudi Arabia; a.alafnan@uoh.edu.sa (A.A.); a.alamry@uoh.edu.sa (A.A.); 2 Department of Pharmaceutics, JSS College of Pharmacy, JSS Academy of Higher Education and Research (JSSAHER), Sri Shivarathreeshwara Nagar, Mysore 570015, India; arvindas76@gmail.com; 3 Department of Pharmaceutics, College of Pharmacy, University of Ha’il, Ha’il 81442, Saudi Arabia; syeddanishpharmacy@gmail.com (S.M.D.R.); afrasimmoin@yahoo.co.in (A.M.); 4 Department of Pharmaceutical Chemistry, College of Pharmacy, University of Ha’il, Ha’il 81442, Saudi Arabia; khushiazeez@yahoo.co.in; 5 Department of Clinical Nutrition, College of Applied Medical Sciences, University of Ha’il, Ha’il 81442, Saudi Arabia; mahgoubamir22@gmail.com (A.M.A.); ao.abdalla@uoh.edu.sa (A.O.E.); 6 Central Manufacturing Technology Institute (CMTI), Tumkur Road, Bangaluru 560022, India; pradyumna@cmti.res.in (P.J.); balashanmugam@cmti.res.in (N.B.); 7 Department of Pharmacognosy, College of Pharmacy, Prince Sattam bin Abdulaziz University, Al-Kharj 11942, Saudi Arabia; drkhalid8811@gmail.com

**Keywords:** microneedle, Projection Micro-Stereo Lithography, preast cancer, fabricated microneedle patches, gemcitabine

## Abstract

Inflammatory breast cancer (IBC) is one of the most belligerent types of breast cancer. While various modalities exist in managing/treating IBC, drug delivery using microneedles (MNs) is considered to be the most innovative method of localized delivery of anti-cancer agents. Localized drug delivery helps to treat IBC could limit their adverse reactions. MNs are nothing but small needle like structures that cause little or no pain at the site of administration for drug delivery via layers of the skin. The polyethylene glycol diacrylate (PEGDA) based MNs were fabricated by using three dimensional (3D) technology called Projection Micro-Stereo Lithography (PµSL). The fabricated microneedle patches (MNPs) were characterized and coated with a coating formulation comprising of gemcitabine and sodium carboxymethyl cellulose by a novel and inventive screen plate method. The drug coated MNPs were characterized by various instrumental methods of analysis and release profile studies were carried out using Franz diffusion cell. Coat-and-poke strategy was employed in administering the drug coated MNPs. Overall, the methods employed in the present study not only help in obtaining MNPs with accurate dimensions but also help in obtaining uniformly drug coated MNPs of gemcitabine for treatment of IBC. Most importantly, 100% drug release was achieved within the first one hour only.

## 1. Introduction

The World Economic Forum’s special report, dated November 2020, identified ‘Microneedles (MNs)’ as one of the top ten emerging technologies for painless injection and blood testing [1]. Generally speaking, MNs are nothing but small needle like structures that cause little or no pain at the site of administration for drug delivery via layers of the skin. They were first conceived and developed in 1957 by Harvey Kravitz and Norman Letvin as a means for vaccination [2]. As a consequence, this concept evolved over a period of time to deliver various active substances for diverse disease conditions. Despite tremendous research efforts in the domain of drug delivery using MNs, more research is focused on developing novel fabrication technologies, optimization of MNs dimension, its insertion to tissues and materials for its fabrication [3,4,5]. As regards the materials, the most popular and biocompatible polymers that are employed include carboxymethyl cellulose [6,7,8], polyvinyl pyrrolidone [9,10], poly glycolic acid, poly lactic acid and poly lactic glycolic acid [11,12,13]. All in all, MNs are set to disrupt the way the drugs are being delivered locally [14]. Hundreds of small needles can be clustered on a small patch, called Microneedle Patches (MNPs) typically of the size of a contact lens for drug delivery. MNPs are designed to penetrate layers of the skin (stratum corneum) to form conduits for diffusion of drugs into dermal circulation and without nerve ending stimulation [8,15,16], thus causing no pain.

**Figure 1:**
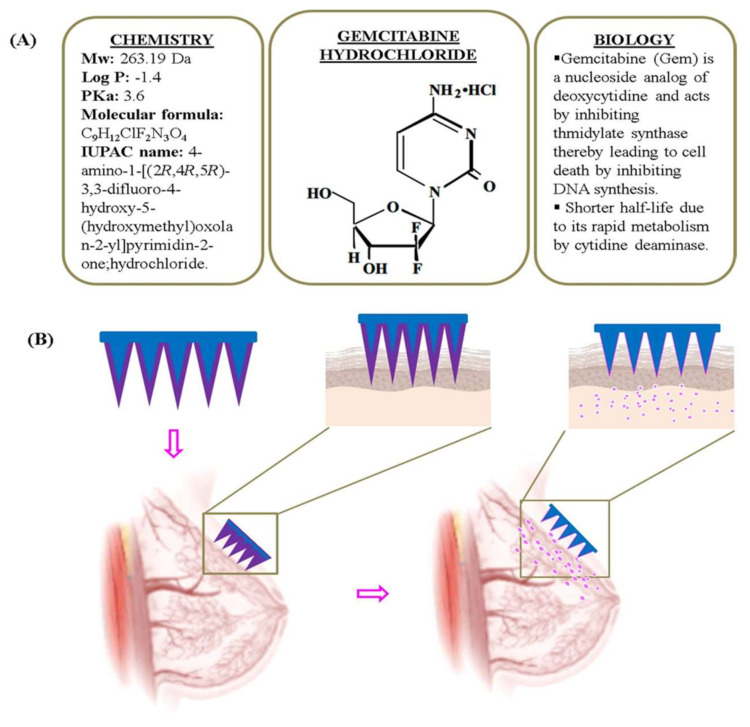
Gemcitabine (Gem) coated microneedles (MNs) (**A**) Gem hydrochloride chemical structure, chemistry and biology (**B**) Coat and poke strategy: insertion of Gem coated MNs and delivery of Gem into the layers of skin.

According to the tumor-node-metastasis (TNM) staging system, Inflammatory Breast Cancer (IBC) is a rare subtype of locally advanced and aggressive form of invasive breast cancer [17]. In general, there are two IBC types—primary and secondary. Primary IBC refers to the development of breast cancer in a normal breast, which eventually transforms into secondary IBC with inflammatory skin changes caused by tumor emboli within the dermal lymphatic ducts [18,19,20]. It is treated using gemcitabine (Gem) either alone or in combination with other agents [21,22], via parenteral route of administration. Figure 1A provides chemical structure of Gem along with its chemical and biological properties.

Major limitations involved in parenteral administration (intravenous delivery) of Gem include shorter half-life (15 to 20 min), severe side effects and development of resistance [23,24,25]. Due to shorter half-life the dose of Gem is usually enhanced which lead to various side effects such as anaemia, neutropenia, myelosuppression, thrombocytopenia and granulocytopenia [26]. So far, research on treatment of IBC has focused primarily on improving systemic therapy. Nonetheless, clinicians have realized the complexity associated with IBC and have mentioned that the prognosis of these patients remain poor [27]. Therefore, there is a need to develop an innovative strategy for drug delivery to treat IBC. MNs based drug delivery could serve as a potential avenue for localized and possibly systemic therapy.

**Figure 2:**
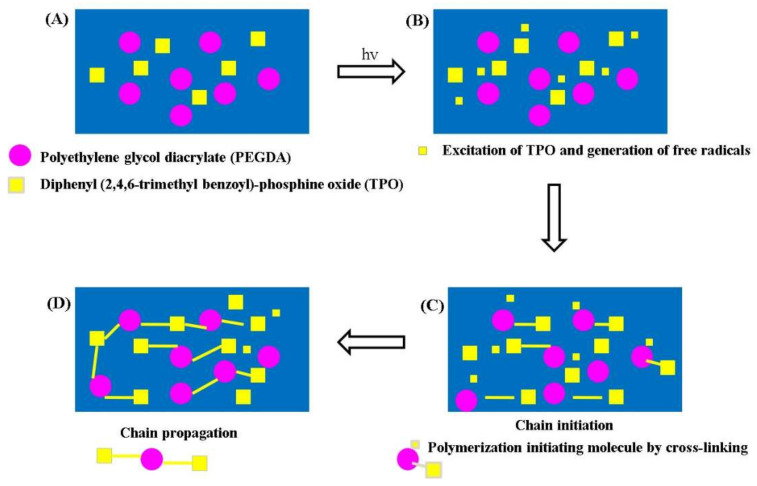
Photo polymerization of polyethylene glycol diacrylate (PEGDA) using diphenyl (2,4,6-trimethyl benzoyl)-phosphine oxide (TPO) as photo initiator (**A**) Monomer and photo initiator (**B**) Generation of free radicals (**C**) Polymerization initiation molecule (**D**) Cross-linking—chain propagation.

Accordingly, the present investigation is aimed at devising and developing polymeric MNPs coated with Gem to treat IBC (Figure 1B), both locally and systemically via transdermal drug delivery. The MNPs of polyethylene glycol diacrylate (PEGDA) were fabricated by a relatively novel three dimensional (3D) micro-fabrication technology called Projection Micro-Stereo Lithography (PµSL) method. More particularly, the study is aimed at developing blank MNs with accurate dimensions by employing proprietary PµSL system, SUKSHM 3D^®^, developed at CMTI [28]. Initially, PEGDA is cross-inked with diphenyl (2,4,6-trimethyl benzoyl)-phosphine oxide (TPO) to form a 3D polymer network. Figure 2 shows the process of PEGDA chain reaction with the photo-initiator TPO. The fabricated blank MNPs were coated with the coating formulation comprising of Gem and sodium carboxymethyl cellulose by a novel and inventive screen plate method. Overall, the methods employed in the present study not only help in obtaining MNPs with accurate dimensions but also help in obtaining uniformly drug coated MNPs of Gem for treatment of IBC.

## 2. Materials and Methods

### 2.1. Materials

Gemcitabine was received as a gratis sample from Shilpa Therapeutics Private Limited, Hyderabad, India. PEGDA (Mw 250 Da), TPO and Parafilm M (laboratory film) were purchased from Sigma Aldrich, St. Louis, MO, USA. PVP-K30 was procured from Himedia Laboratories, Mumbai, India. Porcine skin of about 4 cm in depth was procured from a local butcher shop and was stored in a freezer at a temperature of—25 °C until used. All other chemicals and solvents were of analytical grade. Milli-Q (Millipore, Burlington, MA, USA) water was employed for various experiments in the present study.

### 2.2. Methods-Fabrication of MNPs

#### 2.2.1. Preparation of Polymer Solution

As a first step, PEGDA is combined with ethanol in a ratio of 3:4 (*v*/*v*) followed by mixing under continuous magnetic stirring at a temperature of 200 °C for a time period of 60 min or until a clear solution was obtained. To this, TPO was added (0.25% *v*/*v*) slowly under continuous magnetic stirring for an additional time period of 60 min or until it dissolves completely in the polymer solution. Finally, the vat with photo-curable polymer/resin solution and the *Z*-axis stage (with 50 mm stroke precision, high-resolution Newport Make ILS series) is kept ready and positioned just below the surface of the solution for 3D printing of MNPs.

#### 2.2.2. Loading Modelled MNPs

A 3D computer aided design (CAD) model of MNPs is loaded into the system. The support for MNPs is designed to stabilize the MNs during fabrication. Post-development of CAD files they are converted into STL files which were further sliced into series of cross-sections of desired thickness by the control unit. Thereafter, the image files are supplied as input to the PµSL system (SUKSHM 3D^®^) [28] and was controlled using LabVIEW software NXG 5.0.

#### 2.2.3. Additive Printing by PµSL

**Figure 3:**
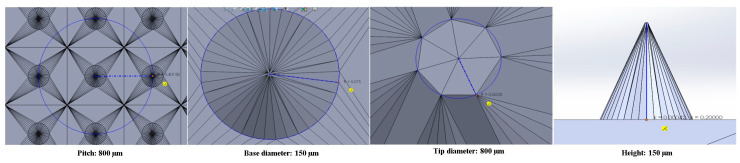
Projection micro-steriolithography (PµSL) for 3D printed microneedles (CAD designs of targeted microneedle dimensions).

The instrument parameters such as UV LED (Luminus, Brussels, Belgium, CBM-120) light intensity, power and projection time parameters were set. A wavelength of 385 nm was employed to fabricate MNPs of the present study. Secondly, the digital micro-mirror device (DMD) is set to process the image files and projection time parameters. Lastly, the thickness of the layer and traverse speed parameters are set. The *Z*-axis stage with detachable silicon substrate is immersed in a photo-curable polymer/resin solution in the vat. Once the printing is started, the stage moves across the axis after printing each layer per each irradiation. The system prints shapes of MNPs as modelled digitally by CAD designing. Post completion of printing, the silicon substrate is detached from *Z*-axis stage and the excess polymer solution, if any, is allowed to drain back into the vat. Isopropyl alcohol is employed as a solvent to rinse the part for removing the remaining uncured photopolymer and a simple air blower is used to blow away excess solvent. After rinsing, the parts were post-cured under UV light for hardening. The set target dimensions of MNPs are pictorially depicted in Figure 3. The cured MNPs were subjected for further characterization by SEM, optical stereo microscope imaging and mechanical characterization methods.

### 2.3. Characterization of MNPs

#### 2.3.1. Stereomicroscopy and Scanning Electron Microscopy

A stereomicroscope (ZEISS SteREo Discovery.V20, Oberkochen, Germany) and a scanning electron microscope (SEM) (Carl Ziess, Neon Crossbeam, Oberkochen, Germany) were used to study the morphology and dimensions of the MNs/MNPs. For SEM, the polymeric MNPs were gold coated (5 to 10 nm) to enhance conductivity.

#### 2.3.2. Mechanical Testing

The blank MNPs were studied for its compression and insertion potential using texture analyser (TA.XT, Stable Micro Systems Ltd., Surrey, UK). For compression studies, Parafilm M (126 µm thick film of waxes and polyolefin’s) was used to simulate skin [29], wherein it was made into eight layers. The method involved insertion of MNPs/array into the film at a speed of 1.19 mm/s, force of 32 N and held for time period of 30 s [29,30]. Needle heights were measured both before and after insertion to the film to determine the compression and are usually expressed as % height reduction. Besides, each layer of the polymeric film was examined microscopically to count the number of holes formed.

### 2.4. Fabrication of Drug-Coated MNPs by Stencil Plate/Screen Method

**Figure 4:**
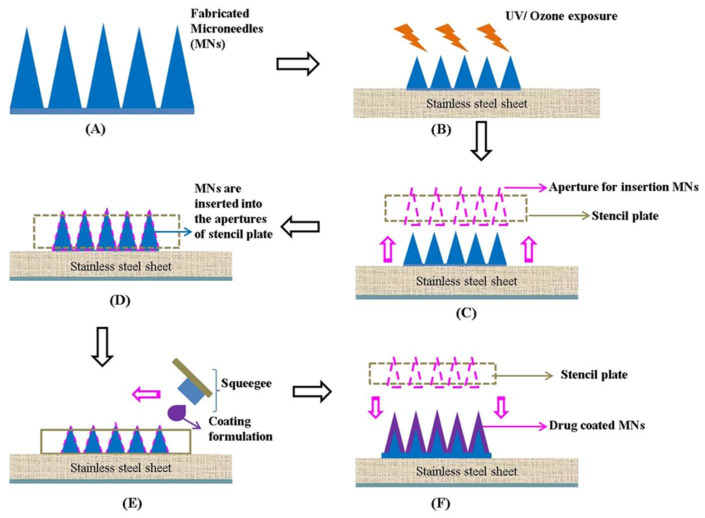
Drug coating of microneedles (MNs) by stencil plate method (**A**) fabricated MNs (**B**) UV/Ozone exposure of MNs (**C**) UV/ozone exposed MNs and stencil plate with apertures (**D**) insertion of MNs into the apertures of stencil plate (**E**) dropping the coating formulation and spreading it with squeegee (**F**) drug coated MNs removal from the stencil plate.

The stencil plate method (Figure 4) of coating MNs comprises steps of initially exposing the blank MNPs to UV/ozone (UV/O3) irradiation for a time period of 2 min using UV/O3 curing system. Secondly, the exposed MNs were inserted into the apertures (5 × 6 = 30 apertures, each having a diameter of 200 µm) of the stencil plate (thickness: 100 µm) followed by dropping (100 µL) the coating formulation comprising of Gem (15% *w*/*w*) and sodium carboxymethyl cellulose (CMC) (8% *w*/*w*) on top of the stencil screen frame, which fills in the apertures as the formulation was spread across the stencil plate using the squeegee at an angle ranging between 75° to 85°. Immediately after coating; the MNPs were air dried for 15 min to ensure firm adhesion of the formulation. The coating process was carried out at a relative humidity of above 70% but below 100%. The final coated MNPs were stored in a desiccator until use for further characterization studies.

### 2.5. Characterization of Drug-Coated MNPs

#### 2.5.1. Fourier Transform Infrared Spectroscopy (FTIR) Analysis

FTIR (FT/IR-4100 Series, Jasco Inc., Easton, MD, USA) spectra were obtained for both the free drug substance (Gem) and drug coated MNPs. Potassium bromide disc method was employed in preparing the drug sample for analysis, wherein the ratio of potassium bromide to drug ratio is 1:1. For drug coated MNPs, three MNs from the MNPs were cut with the help of scalpel followed by mixing with potassium bromide to obtain a pellet. For each spectra, an average of 64 scans were recorded at 4000–500 cm^−1^ at a resolution of 4.0 cm^−1^.

Differential scanning calorimeter (DSC) analysis: DSC (DSC 60, Shimadzu, Kyoto, Japan) was employed to study the thermal transitions of pure Gem and a mixture of Gem and NaCMC.

#### 2.5.2. In Vitro Permeation Studies

Drug permeation studies were carried out through neonatal dermatomed (300 µm) porcine skin (freshly procured from local butchery, Bangaluru, India) by Franz diffusion cells. First and foremost, the porcine skin was cleanly shaved using a razor blade and fixed to the cell donor compartment using glue. The MNPs to be analyzed was inserted at the centre of the skin with gentle pressure for 30 s with a stainless steel weight of 5.0 g on top. The receiver compartment was filled with phosphate buffer saline having pH 7.4 and is thermostatically maintained at a temperature of 37 ± 1 °C. A small magnetic stir bar was used to facilitate agitation (500 rpm) and both the compartments were clamped, with donor compartment on top of the receptor compartment. At predetermined time intervals an aliquot of the sample (200 µL) was collected using syringe from the receiver compartment and replenished with fresh and pre-warmed phosphate buffer saline. The collected samples were filtered, diluted with buffer solvent and vortexed for 20 s followed by HPLC (LC-20, Shimadzu, Japan) analysis. The conditions employed for the analysis are provided in Table 1. The standard Gem solution was prepared in 5, 10, 15, 25 and 30 µg/mL (*n* = 6) concentrations using phosphate buffer to obtain the regression equation (y = 40,525x + 357; r2 = 0.9999). Overall, this method enabled quantification of drug content and expressed as percentage drug released.

### 2.6. Statistical Analysis

The results reported herein are an average with ±standard deviation (*n* ≥ 3). The results were correlated by Student’s *t* test or by ANOVA, *p* < 0.05 was deemed to be significant, unless it is specified. The statistical analysis was carried out using GraphPad Prism, USA.

## 3. Results and Discussion

### 3.1. Fabrication of MNPs

**Scheme 1:**
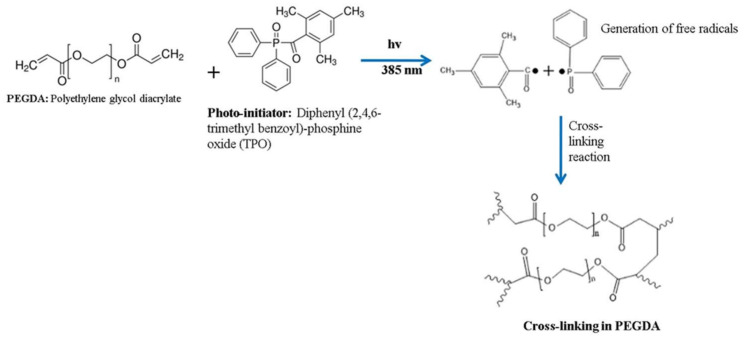
Cross-linking reaction of PEGDA using TPO as photo-initiator under UV LED light exposure.

The MNPs were fabricated by photo-polymerization method using PEGDA as a biopolymer and TPO as a photo-initiator. PEGDA is a popular biocompatible natural polymer characterized by its hydrophilic behavior post polymerization and has the ability to mimic the attributes of an extracellular matrix of a tissue [31]. Polymerization begins when the initiator in the mixture absorbs UV LED radiation at a wavelength of 385 nm to generate free radicals. TPO was selected as a photo-initiator as it shows good absorption at the selected wavelength of 385 nm, though it has higher absorption at short wavelengths. Besides, it also has good solubility in ethanol. More particularly, when TPO absorbs UV LED radiation it undergoes decomposition to generate two free radicals which potentially combine and react with PEGDA (Scheme 1) to initiate the cross-linking reaction, wherein PEGDA chainopening takes place at its carbon-carbon (C-C) bond. The polymer chain initiation is propagated with exiting vinyl bonds on monomers of PEGDA due to which the two chains combine to form a dead polymer chain that terminates the growing polymer chain. The entire photo-polymerization reaction can be controlled by varying various process parameters such as intensity of light, concentration of the photo-initiator or monomer and lastly the exposure time.

The entire fabrication process was carried out using the PµSL system exclusively developed at CMTI and is currently branded as SUKSHM 3D^®^ with a tag-line ‘micro rapid prototyping’ [28]. Primarily, the optical system comprises of UV LED as the light source emitting light in the range of 340 nm to 410 nm, and a peak intensity wavelength of 385 nm. The UV LED light is allowed to pass through a set of optical elements and is projected on the dynamic mask and generates light patterns by aligning several micromirrors according to the projected images as input. Depending on the focal length of the objective lens, the optical module’s reduction factor was changed. In short, if the focal length is fixed the reduction factor value also can be fixed at a particular value. The prime focus of the present study was to fabricate dimensionally accurate MNPs using SUKSHM 3D^®^ system, wherein it systematically corrects the optical reduction ratio by employing suitable processing parameters (not shown here). The optical reduction factors of the system were calculated (not shown here) and examined for dimensional inaccuracies using a stereomicroscope. The percentage of the error in the dimensions of fabricated MNs was found to be about 1.375%. Accordingly, the SUKSHM 3D^®^ system helped obtain MNPs with accurate dimensions as expected (but with minor variations), which are discussed below.

### 3.2. Characterization of MNPs

#### 3.2.1. Stereomicroscopy and Scanning Electron Microscopy

The fabricated blank MNPs were about 1 mm in size comprising of a total of 30 (6 by 5) MNs per patch in conical form. The obtained dimensions of MNs over the set dimensions are shown in Table 2.

**Figure 5:**
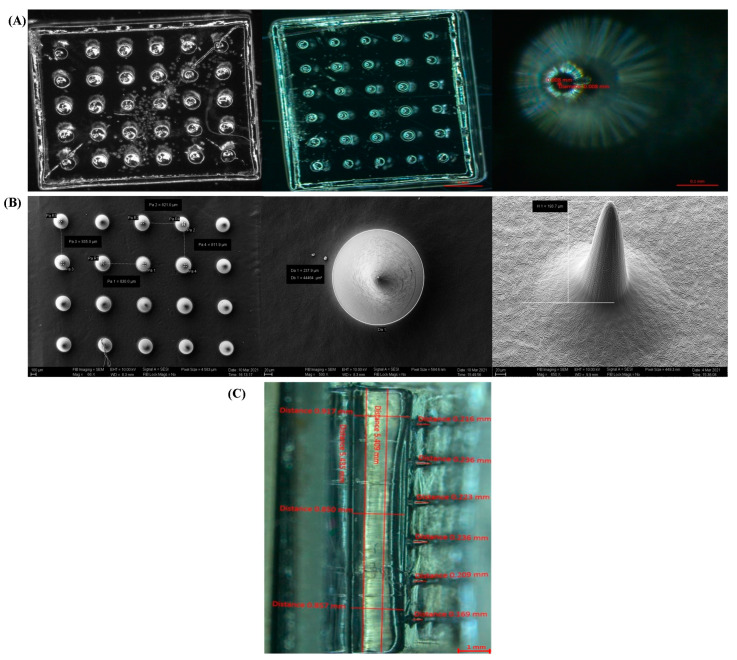
Dimensions of MNs (**A**) Stereomicroscopic images (**B**) Scanning electron microscopy images (**C**) Complete dimensions of a single fabricated microneedle patch in millimetres (mm).

The MNs on the MNPs were perpendicular to the patch base. Figure 5A shows optical stereomicroscopic images of fabricated MNPs. Similarly, Figure 5B shows pitch of the MNs, the total base diameter, which is 237.9 µm and height of a single MN as 193.7 µm. Figure 5C shows complete characterization of the dimensions of single MNPs.

Overall, the dimensions of fabricated MNs base and tip diameter and pitch were slightly higher than the set target. On the other hand, the height of the MNs was slightly less than the targeted dimensions. Nonetheless, the fabricated MNs were found to be acceptable for the proposed study.

#### 3.2.2. Mechanical Testing

Turning now towards the mechanical testing of blank MNPs, mechanical strength of MNs is a very critical parameter as they need to pierce the skin layer (stratum corneum) to deliver the drug without pain. Mechanical strength, particularly the effect of compression was determined using TA.XTS texture analyzer. A uniform and optimum force of 32 N per each MNPs for 30 s was employed for the study. These parameters were also employed by previous researchers as they simulate the natural force employed by humans while applying MNPs to subjects in need thereof [30,32]. The compression analysis was carried out along with the insertion study using eight film layers. More than 30% of the needles penetrated 3rd layer and less than 30% penetrated the 4th layer. Larraneta et al. described that a penetration is considered as successful when the number of holes created on each film layer is more than 20% [30]. Accordingly, it was deduced that the MNPs of the present study were lying between the third and fourth film layers [33,34].

### 3.3. Fabrication of Drug-Coated MNs by Stencil Plate/Screen Method

MNs have gained tremendous attention due to its potential to overcome the thick stratum corneum layer of the skin by piercing it to deliver drugs into the microcirculation of epidermis and dermis. Post insertion into the layers of skin and depending on the type of strategy employed the MNs interact with the interstitial fluid and dissolves to release the drug into the layers of epidermis or upper dermis [32,35]. The present study employed ‘coat and poke’ strategy, wherein the coating formulation is diffused from the surface of MNs into the layers of the skin [2]. Nonetheless, uniformity in coating is critical for effective drug delivery. In addition, inefficient coating could lead to decrease the sharpness of MNs. To fixthis issue, the present study employed a novel and inventive set-up to have uniform drug coating on microneedle surface for effective drug release without compromising on the sharpness of the MNs.

Before coating, the blank MNs were exposed to UV/O3 curing system, wherein the UV energy helps break the covalent bonds in the polymer and ozone help oxidize the polymer surface thereby converting the hydrophobic polymer surface to a more hydrophilic surface for efficient drug coating and release profiles [36]. As regards the coating formulation, CMC is employed as a viscosity enhancing agent for the drug solution to coat on MNs and to prevent it from flowing down during the drying stage. The stencil plate method when used, the 8% (*w*/*w*) solution of CMC coated 100 to 150 µg of Gem on the surface of MNs. Nonetheless higher percentage of CMC helped achieve low concentration of Gem coating on MNs. This was probably due to significant increase in viscosity (>3000 cp) with increase in concentration of CMC (>8% *w*/*w*), thereby leading to irregularity in wetting the MNs. Therefore, 8% (*w*/*w*) CMC was considered to be ideal for drug coating the MNs.

### 3.4. Characterization of Drug-Coated MNPs

#### 3.4.1. FTIR Analysis

**Figure 6:**
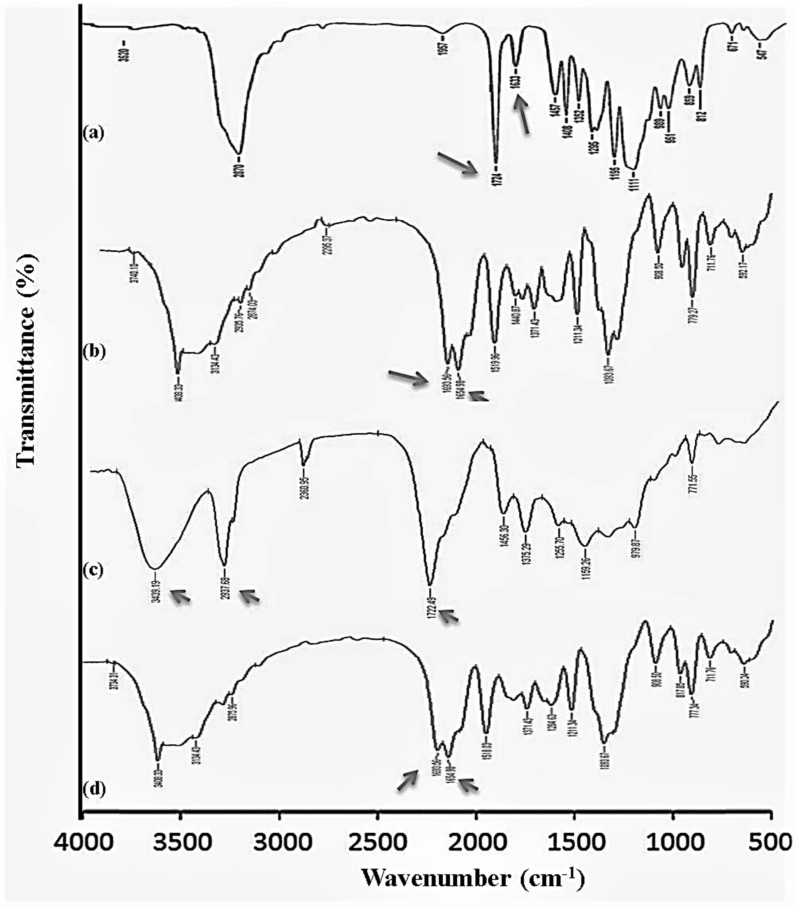
FTIR spectrum (**a**) Polyethylene glycol diacrylate (PEGDA) (**b**) gemcitabine (**c**) sodium carboxymethyl cellulose (NaCMC) (**d**) mixture of gemcitabine and NaCMC.

FTIR analysis was carried out to assess the interaction, if any between the components of drug coated MNPs. PEGDA is a derivative of polyethylene glycol and has good properties such as flexibility, non-toxic, non-immunogenic and hydrophilic. It has acrylic groups on either side of its chain which are ready to undergo polymerization process. It’s FTIR spectrum (Figure 6a) shows peaks at 1633 cm^−1^ and 1724 cm^−1^ corresponds to C=C (aliphatic double bond) and C=O (carbonyl group). The FTIR spectrum of the drug substance (gem) (Figure 6b) showed characteristic peaks at 1693 cm^−1^ for amine band, 1654 cm^−1^ for amide group and 3408 cm^−1^ for amino stretching vibration. Similarly, the FTIR spectrum of sodium carboxymethyl cellulose (NaCMC) (Figure 6c) showed peaks at 1722 cm^−1^ for C=O, 2937 cm^−1^ for C-H stretch and 3439 cm^−1^ for N-H stretch. The spectra (Figure 6d) of the physical mixture (gem and sodium carboxymethyl cellulose) showed peaks at similar wave numbers. The visual inspection of each spectrum (Figure 6) did not show any shift in peaks. Accordingly, it was proving that no interactions found between the components of MNPs.

#### 3.4.2. DSC Analysis

**Figure 7:**
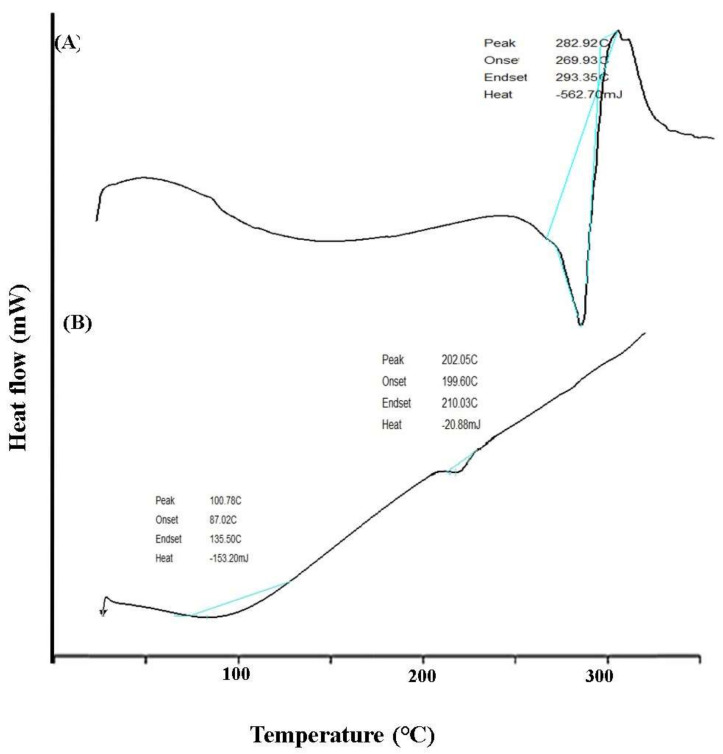
DSC thermograms of (**A**) pure gemcitabine and (**B**) mixture of gemcitabine and sodium carboxymethyl cellulose.

The DSC thermogram (Figure 7) shows a sharp peak of 282.9 °C which corresponds to the melting point of the drug. Whereas the mixture of gemcitabine with sodium CMC shows the disappearance of the peak indicating the molecular dispersion of the drug. Other than this no other effects were observed indicating drug polymer compatibility.

#### 3.4.3. In Vitro Permeation Studies

Insertion of MNs into the porcine skin was ensured before proceeding with in vitro permeation study. MNPs were carefully inserted into the porcine skin (1 mm thickness) with constant finger pressure for 30 s. Further confirmations were made by visual microscopy using optical coherence tomography (EX 1301 OCT). The drug coated MNs had the potential to bypass the stratum corneum and reach the dermis. The amount of drug coated onto the blank MNs was 105.05 ± 4.5 µg with negligible amount (<2 µg) found on the base of MNPs.

**Figure 8:**
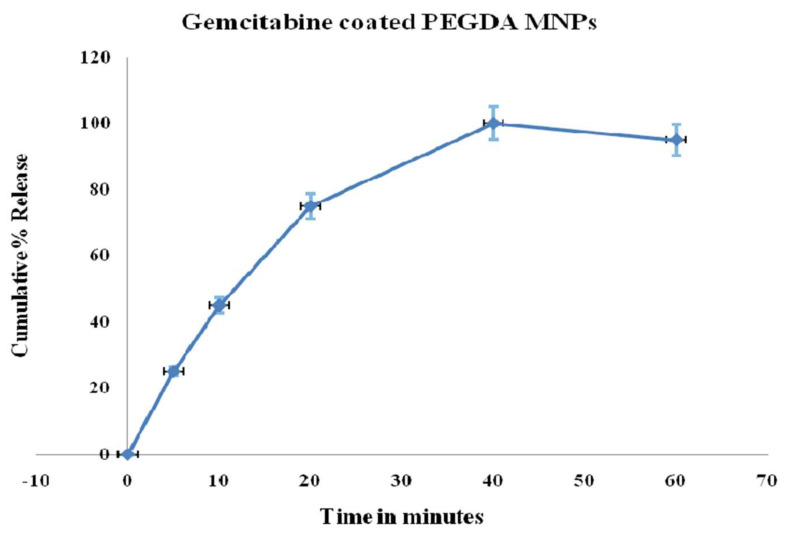
Gemcitabine release profile after coating on microneedles. Release studies were carried out by using Franz diffusion cell. Data presented as mean ± SD, *n* = 3.

After 5 min, HPLC analysis confirmed detection of Gem in the receiver compartment of Franz diffusion cell. As illustrated in Figure 8, 100% of Gem was released from coated MNs within the first one hour. Besides, only minor percentage (5%) was released after 48 h.

## 4. Conclusions

The PEGDA MNs were fabricated with accurate dimensions using PµSL system. Moreover, it also demonstrates that efficient drug coating of MNPs is possible using screen plate method. Overall, the Gem coating on PEGDA MNPs has enabled drug delivery in vitro and could be employed for treatment of IBC. More in vivo studies are required to study the effectiveness of the drug delivery.

## Figures and Tables

**Table 1:** HPLC Conditions for determination of Gem recovery from MNPs.

Item	Details
Mobile phase	Phosphate buffer pH7.4 and methanol in 90:10 ratio
Injection volume	40 µL
Flowrate	1 mL/min
Absorbance wavelength	275 nm (UV-Visible detector)
Column temperature	10 °C
Column	C18(250X4.6mmi.d., 5 µm, Phenomenex, Torrance, CA, USA)

**Table 2:** Dimensions of microneedles.

Sl. No.	Item	Target Dimensions (µm)	Average of Obtained Dimensions (µm)
1	Base diameter	150	158
2	Tip diameter	5	8
3	Height	200	193
4	Pitch	800	824

## Data Availability

Data is contained within the article.
